# Downregulated Expression of Peroxiredoxin 4 in Granulosa Cells from Polycystic Ovary Syndrome

**DOI:** 10.1371/journal.pone.0076460

**Published:** 2013-10-03

**Authors:** Yan Meng, Yi Qian, Li Gao, Ling-Bo Cai, Yu-Gui Cui, Jia-Yin Liu

**Affiliations:** The State Key Laboratory of Reproductive Medicine, Clinical Center of Reproductive Medicine, the First Affiliated Hospital, Nanjing Medical University, Nanjing, Jiangsu Province, China; Institut Jacques Monod, France

## Abstract

Peroxiredoxin 4 (PRDX4), a member of Peroxiredoxin (PRDX) family, is a typical 2-Cys PRDX. PRDX4 monitors the oxidative burden within cellular compartment and reduces hydrogen peroxide and alkyl hydroperoxide related to oxidative stress and apoptosis. Antioxidant, like PRDX4, may promote follicle development and participate in the pathophysiology of PCOS. In our previous study, we found that PRDX4 was expressed in mice oocyte cumulus oophorus complex, and that PRDX4 could be associated with follicle development. In this study, we explored the expression of PRDX4 in human follicles and possible role of PRDX4 in PCOS pathophysiology. Our data showed that PRDX4 was mainly expressed in granulosa cells in human ovaries. When compared to control group, both PRDX4 mRNA level and protein level decreased in PCOS group. The lowered levels of PRDX4 may relate to oxidative stress in the pathophysiologic progress of PCOS. Furthermore, expression of PRDX4 in the granulosa cells of *in vivo* or *in vitro* matured follicles was higher than that in immatured follicles, which suggested that PRDX4 may have a close relationship with follicular development. Altogether, our findings may provide new clues of the pathophysiologic mechanism of PCOS and potential therapeutic strategy using antioxidant, like PRDX4.

## Introduction

Polycystic ovary syndrome (PCOS), one of the most common female endocrine disorders affecting about 5–10% women of reproductive age, is a major cause of anovulatory infertility [1]. Its etiology and detailed pathophysiology remain uncertain. Although clinical and biochemical features of PCOS are typically heterogeneous [2], [3], abnormal folliculogenesis is considered as a common characteristic. Hughesdon *et al* observed that ovaries of PCOS patients contained twice higher number of growing follicles at every development stage than control group [4]. The aberrant progression of follicular development through the earliest stage of folliculogenesis was found in ovaries from anovulatory women with PCOS [5]. There was an increased density of primary follicles in PCOS ovaries when compared to normal ovaries, which suggested the errant folliculogenesis in PCOS. It may be the main cause of anovulation and other metabolic disturbances [6]. It was concluded that disorders of follicular development in PCOS were closely related to apoptosis of granulosa cells (GCs) which was associated with the increased oxidative stress(OS)/reactive oxygen species (ROS) generation [7], [8]. In the environment of oxidative stress caused by an imbalance between pro-oxidants and antioxidants, more GCs suffered apoptosis, which showed a lower protection to oocytes [9]. While this procedure could have negative effects on the clinical outcomes of *in vitro* fertilization (IVF) in PCOS patients [7], [10], [11].

Antioxidant is a family of proteins preventing cells from OS/ROS damage.The apoptosis index of ovarian GCs cultured *in vitro* was lowered by adding antioxidants into the culture media, which was favored to oocyte development [8]. PRDX4, a secretory antioxidant of the peroxiredoxin family, is expressed in various organisms [12]. There are at least six distinct PRDX genes (PRDX 1–6) in human, only PRDX4 is localized in the endoplasmic reticulum (ER) [13]. Accumulated data indicated that the PRDX family is associated with female reproduction and PRDX2 is detected in human follicular fluid [14]. PRDX6 expression levels were increased in bovine oocytes and cumulus cells during in vitro maturation (IVM) [15]. However, the role of PRDX4 in ovary is unclear since there are few reports on the PRDX4 in female reproductive system. As an antioxidant protein, PRDX4 can reduce intracellular ROS, specifical peroxides such as hydrogen peroxide, thereby influencing the signaling pathways related with intracellular oxidation. This functional peroxidase activity is dependent on the reduced forms of thioredoxin and/or glutathione [16]. In our previous study, PRDX4 was found to be expressed in mice oocyte-cumulus oophorus complex (COC) [17]. Compared with mouse small follicles, those large follicles had the increased expression of Prdx4, suggesting that PRDX4 may play a role in follicle development. This study was designed to explore the expression of PRDX4 in human normal and PCOS ovaries and its role in follicular development, and to discuss possible roles of PRDX4 in the PCOS pathogenesis.

## Materials and Methods

### Subjects

The ovarian samples of fifteen women were collected during transsexual operation and used as controls. The hormonal treatment was withdrawed at least 3 months in those transsexual patients before operation. The ovarian samples of fifteen PCOS patients were collected during surgical treatment. The diagnosis of PCOS was according to the revised Rotterdam European Society of Human Reproduction and Embryology/American Society for Reproductive Medicine Criteria [18]. All PCOS women had oligomenorrhea or amenorrhea (eight or fewer spontaneous menses per year), clinical (hirsutism), and/or biochemical (elevated free androgen index) evidence of hyperandrogenism and polycystic ovaries by ultrasound scanning [19]. This study was approved by the Institutional Ethics Committee of The First Affiliated Hospital of Nanjing Medical University. All volunteers gave the signed consent forms.

Five samples of PCOS ovaries and five samples of control group were fixed in 4% Paraformaldehyde for 24 h, stored in 70% ethanol, and dehydrated and embedded in paraffin. The paraffin-embedded samples were used for pathology diagnosis and immunohistochemical staining. Ten fresh ovarian tissues of PCOS and control group, respectively, were snap frozen and stored in liquid nitrogen until five of them were used for RNA extraction and qRT-PCR, while the other five samples were used for protein extraction and Western blot analysis. The Western blot and qRT-PCR experiments were repeated four times.

### Granulosa Cells

GCs were isolated from 16 women with PCOS undergoing *in vitro* fertilization and embryo transfer (IVF-ET) patients. 26 women with regular menstrual cycles were enrolled in control group, who were diagnosed as tubal or male factor infertility. The detailed medical history was taken. The volunteers were ≤32 years old, had body mass index ≤23 kg/m^2^, and had no history of other gynecological or medical disorders. All subjects were injected with a GnRH agonist –Lupride (Sun Pharmaceuticals, Mumbai, India) starting from mid-luteal phase. Once pituitary down-regulation was achieved after two weeks, they were injected with recombinant FSH (Gonal F, Serono, Geneva, Switzerland). Follicular size was monitored regularly by ultrasound and serum estradiol assays. When there were three or more follicles with mean diameter ≥17 mm, hCG (Pregnyl, Organon, The Netherlands) was administered subcutaneously. At 34–36 h following the administration of hCG, follicle size was estimated under ultrasound and then oocytes were aspirated. Follicular fluid of small follicles [(SFs, <12 mm)] and large follicles [(LFs, ≥16 mm)] from the same patient were collected in separate tubes. SFs and LFs were separately represented for immature and mature follicles [20].

### *In Vitro* Maturation for PCOS Immature Oocytes

Sixteen PCOS women undergoing in vitro maturation (IVM) were enrolled. All women were primed with 75 IU recombinant FSH daily initiated on cycle day 3. Immature oocyte retrieval was scheduled in midfollicular phase (cycle day 9 to 11). Patients were given one subcutaneous injection of 6500 IU of hCG when the endometrial thickness reached 6.0 to 8.0 mm and the largest follicle was not bigger than 10 mm in diameter. Oocyte retrieval was performed 36 hours after hCG injection [21]. The maturity of oocyte was assessed after 24 hours of IVM culture based on the presence of the first polar body in perivitelline space. The GCs around oocytes after oocyte in vitro maturation were collected for further study.

### Isolation of Granulosa Cells

Follicular aspirates included both oocytes and GCs. After removal of the oocyte, follicular aspirates of SFs or LFs from the same patient were centrifuged at 2000×g for 10 min and the supernatant were slowly removed using Pasteur pipette. Pellet was resuspended in 1 ml of Dulbecco’s Modified Eagle Medium (DMEM) and gently layered on the top of 2 ml 50% Percoll density gradient and centrifuged at 800×g for 10 min. After centrifuging, GC layer will be located between Percoll and DMEM medium. These cells were then collected and washed twice in 3 ml of DMEM medium (centrifuged at 1500×g for 5 min each time). Furthermore, red blood cells were removed by using red blood cell lysis buffer. Ten of the twenty-six control group paired samples were prepared for Western blot. Two samples were pooled together to get enough cells and the experiment was repeated four times. Sixteen GC samples from PCOS, control and IVM groups were stored for qRT-PCR, every four samples were pooled to get enough cells and the experiment were independently repeated four times. This study was approved by the Institutional Ethics Committee of The First Affiliated Hospital of Nanjing Medical University.

### Immunohistochemical Analysis

Ovarian tissues were processed and cut into 5 µm sections for IHC according to previous study [22]. Sections were incubated with Rabbit anti-mouse PRDX4 antibody (1∶800 dilution, Ab59542, Abcam, Cambridge, UK) overnight at 4°C. Immunoreactive sites were visualized with diaminobenzidine (DAB) (ZLI-9032, Zhongshanjinqiao, China) and mounted for bright field microscopy (Axioskop 2 plus, Zeiss, Germany). A negative control slide was processed exactly the same way, but using rabbit IgG as primary antibody (1∶800 dilution, SC-2027, SANTA, Texas, USA).

### Western Blot Analysis

Total proteins from ovaries and granulosa cells were prepared using lysis buffer and quantified by the BCA method (Biyuntian, Shanghai, China). Rabbit anti-mouse PRDX4 antibody (1∶800, Ab59542, Abcam, Cambridge, UK) and rabbit anti-GAPDH antibody (1∶7000 dilution, Ab8245, Abcam, Cambridge, UK) were used for Western blot, performed as previously described [23].

### RNA Extraction and qRT-PCR

Total RNA was isolated from the collected GCs using Trizol reagent (Invitrogen, Carlsbad, CA, USA), according to the manufacturer’s instructions. Reverse transcription was performed using Superscript III reverse transcriptase (Invitrogen, Carlsbad, CA, USA). RNA purity was measured using 260/280 nm absorbance ratio. The integrity of the RNA preparations was evaluated by electrophoresis on 2% agarose gels containing 0.005% goldview (Shanghai SaiBaiSheng, Shanghai, China). RNA with intact ribosomal 28S and 18S RNA bands, with an intensity ratio of 1.0–1.5, was used for qRT-PCR. cDNAs were amplified using QuantiTect SYBR Green PCR kits (Takara Shuzo Co Ltd, Kyoto, Japan) and the level of mRNA for PRDX4 gene relative to GAPDH was calculated using the ΔΔCT method [24]. Primers for PCR are following:

GAPDH: sense: 5′-GAAGGTCGGAGTCAACGGATTT-3′;

antisense: 5′-CTGGAAGATGGTGATGGGATTTC-3′;

PRDX4: sense: 5′-AGAGGAGTGCCACTTCTACG-3′;

antisense: 5′-GGAAATCTTCGCTTTGCTTAGGT-3′.

### Statistical Analysis

Statistical evaluations were performed using SPSS Software (version 16.0; SPSS, Inc., Chicago, IL, USA). All data were shown as mean ± SEM and analyzed using Students t-test. *p*<0.05 was considered significant; *p*<0.01 was considered highly significant.

## Results

### Expression of PRDX4 in Human Ovaries

**Figure 1:**
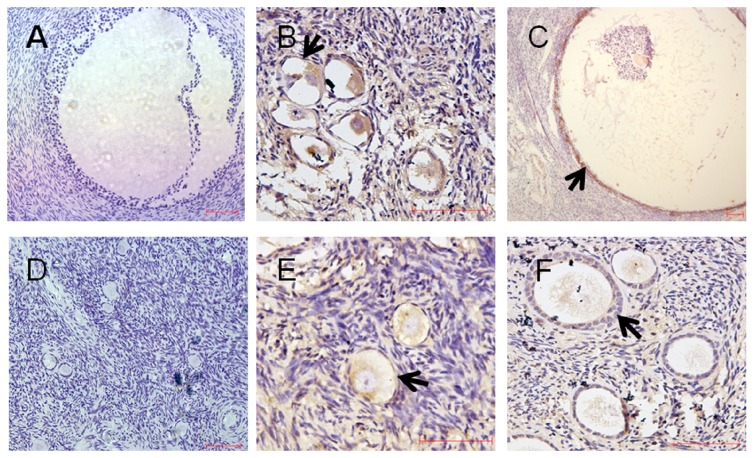
Expression of PRDX4 protein was mainly located in granulosa cells of human ovaries. Cellular localization of PRDX4 expression was evaluated in normal ovaries and PCOS ovaries. Five samples of PCOS ovaries and five samples of control group were fixed in 4% Paraformaldehyde for 24 h, replaced in 70% ethanol, dehydrated and embedded in paraffin. The paraffin-embedded samples were used for pathology and immunohistochemical staining. Sections were incubated with anti-PRDX4 antibody. A, B, C were normal ovarian tissues. D, E, F were PCOS ovarian tissues. Panel A, D were negative control slides,using IgG as primary antibody. Expression of PRDX4 protein was most prominent in granulosa cells in both two groups. Arrows indicated positive immunoreactive signals in granulosa cells visualized as brown staining. Scale bar = 50 µm.

To observe the localization of PRDX4 protein in human ovaries, we used immunohistochemical analysis in five normal and five PCOS ovarian tissues. As indicated by the results (Figure 1), expression of PRDX4 protein was mainly located in granulosa cells of both normal (B and C) and PCOS ovarian tissues (E and F).

### PRDX4 Decreased in PCOS

**Figure 2:**
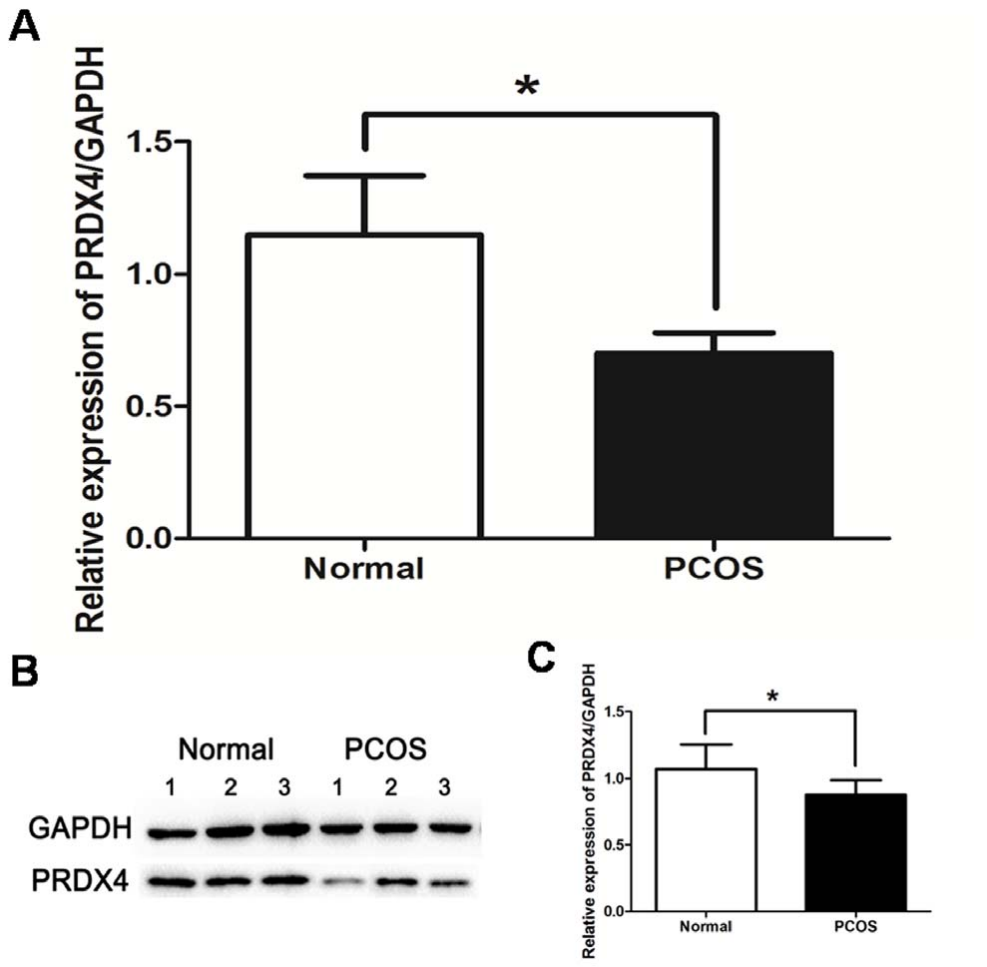
Expressions of PRDX4 mRNA and protein in normal ovaries and PCOS ovaries. Total RNA and total protein were individually extracted from five separate ovarian tissues of normal and PCOS groups. Expression level of PRDX4 mRNA in control group were twice as high as that in PCOS group (p<0.05) (A). Consistent with above results, the Western blot results indicated that expression level of PRDX4 protein was overall lower in PCOS group compared to the control group (p<0.05) (C). And three representative Western blot results of each group were presented (B). The experiments were repeated four times. Data were presented as mean ± SEM. *, p<0.05.

Western blot and qRT-PCR were used to quantify the differential expression of PRDX4 between control and PCOS groups. qRT-PCR results showed that expression level of PRDX4 mRNA in control group were twice as high as that in PCOS group (*p*<0.05) (Figure 2A). Consistent with the qRT-PCR results, the Western blot results indicated that protein level of PRDX4 was overall lower in PCOS compared to the control group (Figure 2B and C).

### PRDX4 had a Higher Expression in Granulosa Cells of Mature Follicles

**Figure 3:**
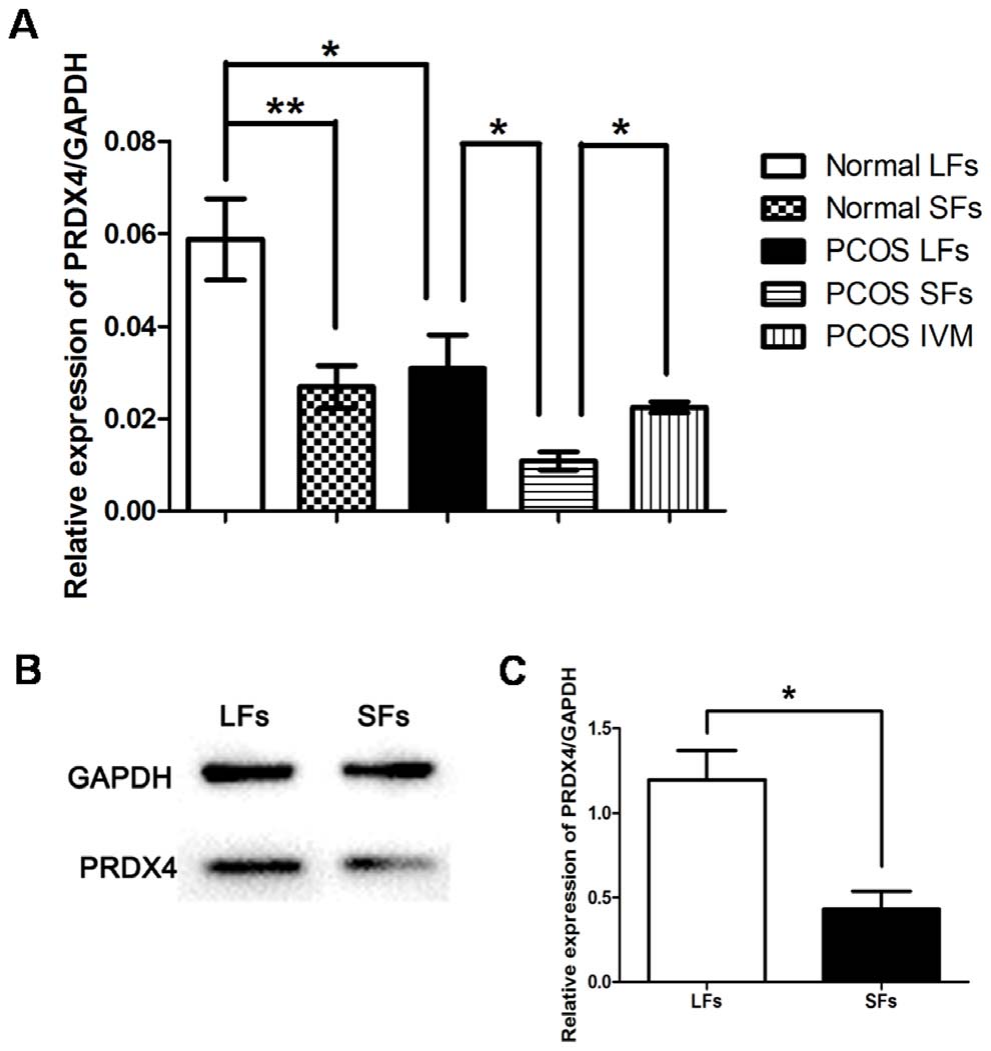
Different expression of PRDX4 protein and mRNA in LFs (mature follicles) and SFs (immature follicles). Expression level of PRDX4 mRNA in granulosa cells were detected by qRT-PCR (A). Sixteen GCs samples from PCOS group and Sixteen from control group were used, every four samples were pooled to get enough cells. Expression level of PRDX4 mRNA in LFs was much higher than that in SFs, almost two fold in both normal group (A, Normal LFs *vs.* Normal SFs) and PCOS group (A, PCOS LFs *vs.* PCOS SFs) (p<0.05). To detect the change of expression of PRDX4 mRNA during IVM, granulosa cells from sixteen IVM patients were obtained. Expression level of PRDX4 mRNA was incresed after IVM (A, PCOS IVM *vs.* PCOS SFs), the same chang was found after *in vivo* maturation (A, PCOS IVM *vs.* PCOS LFs). Ten samples of control group were used in Western blot, every two samples were pooled to get enough cells. The representative data were showed in this figure (B). GAPDH was used as the standardized reference. PRDX4 was differently expressed in the granulosa cells of normal ovarian LFs and SFs. Expression level of PRDX4 protein in granulosa cells of LFs was almost twice as high as that in SFs (p<0.05) (C). The experiments were singly repeated four times. Data were presented as mean ± SEM. *, p<0.05; **, p<0.01.

The differencial expressions of PRDX4 in SF (immature follicle) and LF (mature follicle) granulosa cells were compared. Expression of PRDX4 mRNA in LFs was much higher than that in SFs, with almost two fold in both normal and PCOS groups (p<0.05) (Figure 3A, Normal SFs vs. LFs and PCOS SFs vs. LFs). Furthermore, Western blot results demonstrated that PRDX4 was differently expressed in granulosa cells of normal ovary SFs and LFs (Figure 3B). It was found that expression of PRDX4 protein was also almost two fold in LF GCs compared to SF GCs (p<0.05) (Figure 3C). These data strongly demonstrated that PRDX4 had a possible role in regulating follicular development and maturation.

### Expression of PRDX4 in PCOS Granulosa Cells Undergoing IVM

Expression of both PRDX4 protein and mRNA decreased in PCOS granulosa cells compared with control groups. Furthermore, we obtained the *in vitro* matured granulosa cells from sixteen PCOS patients undergoing IVM program. qRT-PCR data indicated that expression of PRDX4 mRNA increased almost two fold higher in the matured follicle GCs after IVM than immature GCs, the difference was significant (p<0.05) (Figure 3A, PCOS IVM vs. PCOS SFs), and nearly the same level as in vivo matured follicle GCs of PCOS patients (p>0.05) (Figure 3A, PCOS IVM vs. PCOS LFs).

## Discussion

In this study, we reported for the first time that PRDX4 was closely associated with pathophysiology of PCOS. Our data indicated that PRDX4 protein was mainly expressed in human GCs, and that expressions of both PRDX4 protein and PRDX4 mRNA lowered in PCOS ovaries compared with normal control. Secondly, we found that expression of PRDX4 was associated with follicular development. Expression of PRDX4 in LFs was higher than that in SFs, both in normal ovaries and PCOS ovaries, while expression of PRDX4 in the PCOS granulosa cells undergoing both in vitro and in vivo maturation significantly increased when compared with granulosa cells of immature COCs.

PRDX4 exerts protective function against oxidative damage by scavenging reactive oxygen species in human tissues [25], [26]. The peroxidatic activity of PRDX4 in cellular ER was shown to be important for removing H_2_O_2_ produced by Ero1 during disulfide formation, playing an important role in dampening H_2_O_2_ levels in the ER [27]. The antioxidant function of PRDX4 has been demonstrated in different models. The PRDX4 deficient mice exhibited clear increases of oxidative stress markers (8-oxo-dG and HNE), which was accompanied by testicular cell abnormalities [28]. Knockdown of PRDX4 in human HT1080 cells resulted in the reduced cell viability when challenged with H_2_O_2_ [29]. PRDX4, as an antioxidant, play a role protecting cells from the oxidative stress damage caused by ROS in acute promyelocytic leukemia and lung cancer [30], [31].

Although the exact etiology and pathogenesis of PCOS still remain obscure, aberrant follicular development, a common characteristic of PCOS, is closely associated with GCs apoptosis caused by oxidative stress [32]. Oxidative stress may be induced by the increased levels of reactive oxygen species, or the decreaseed antioxidant defense mechanisms [33], [34]. Victor et al found that there was a decrease in mitochondrial O_2_ consumption and GSH levels along with an increased ROS production, suggesting the mitochondrial dysfunction in PCOS patients [35]. A meta-analysis in 2013, including 68 clinical studies, showed a presence of elevated concentrations of oxidative stress markers in patients with PCOS [36]. All above studies indicated that PCOS was a disease associated with the decreased antioxidant concentrations, an oxidative state [37]. In this study, we found that PRDX4 protein was predominantly expressed in granulosa cells of human ovarian tissues, and that the amounts of PRDX4 protein and mRNA expressions in PCOS ovaries were only half of those in normal ovaries. The lack of antioxidant PRDX4 may be closely related to cellular responses of follicular development and pathophysiology of PCOS. In human cell lines, PRDX4 was identified either as a cytoplasmic protein attenuating NF-kappa B activation [26], or as a secretable protein activating both NF-kappa B and JNK [38], both of which were involved in apoptosis of GCs and the pathologic progress of PCOS [39], [40].

Nowadays, more evidence showed that the increased formation of oxidized molecules and reactive oxygen species within ovaries played a crucial role in the initiation and progression of follicular development. Expressions of various oxidative stress biomarkers were detected in normally functioning human ovaries [41]. ROS served as key signal molecules in a variety of physiological processes from oocyte maturation to fertilization, pregnancy, and embryo development in female reproductive processes [42], in which accumulation of OS/ROS may play a negative role. In this study, we found the increased expression of PRDX4 in mature follicles compared to immature follicles (Figure 3) which was consistent with our previous study [17]. Related to ovulation, ROS concentration increased in large follicles compared to small ones [43], which may explain the increase of PRDX4 with follicular development. When the immature PCOS follicles were cultured *in vitro*, ROS in the environment stimulated PRDX4 expression to protect GCs from oxidative stress injury. This may explain the increased PRDX4 expression in GCs after IVM.

**Figure 4:**
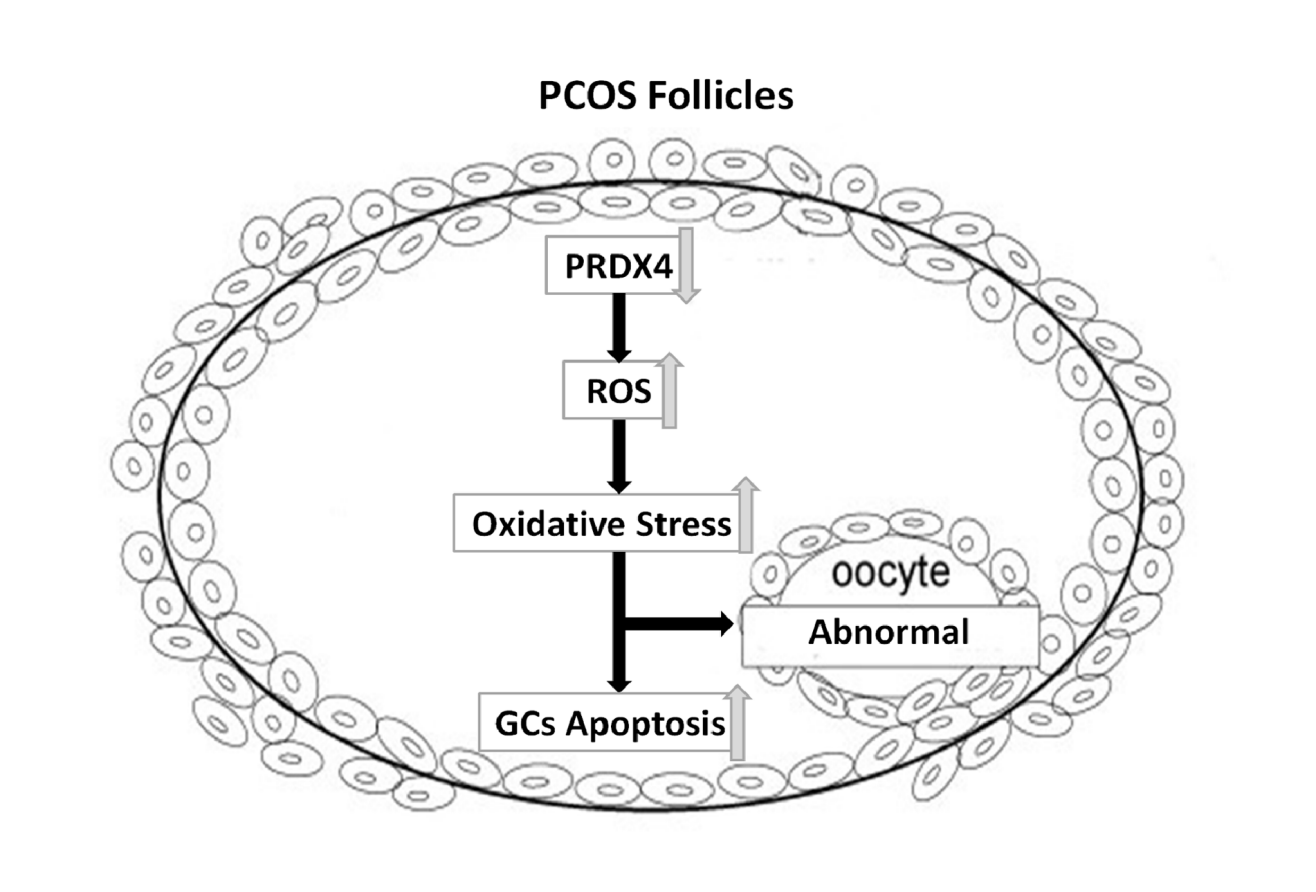
Schematic model illustrated the possible roles of PRDX4 in PCOS ovarian follicles. PRDX4, as an antioxidant, mainly located in granulosa cells, can partly counteract ROS in follicles. The lowered expression of PRDX4 in PCOS ovaries may contribute to the oxidative stress related to granulosa cell apoptosis and follicular development abnormality.

In this study, it was suggested that expression of PRDX4 in PCOS ovaries appeared to be mediated through oxidative stress in GCs. We reported that the deficiency of antioxidant PRDX4 was associated with pathophysiological mechanism of PCOS. PRDX4, as an antioxidant, located mainly in granulosa cells, counteracts ROS in follicles. The lowered expression of PRDX4 in PCOS ovaries may contribute to the oxidative stress related to apoptosis of granulosa cell and developmental abnormality of follicles (Figure 4). Anyway, the exact pathways need more study in future.
